# Dataset of numerical simulations for aeroelastic control of an aero engine compressor cascade using plasma actuators

**DOI:** 10.1016/j.dib.2021.107584

**Published:** 2021-11-16

**Authors:** Maria Grazia De Giorgi, Valentina Motta, Antonio Suma, Alessia Laforì

**Affiliations:** a Department Engineering for Innovation, University of Salento, Via per Monteroni, 73100 Lecce, Italy; b General Electric, Advanced Technology & Sciences, Franklinstraße 14, 10587 Berlin, Germany

**Keywords:** Load alleviation, Cascade, Flutter, AC-DBD plasma, Flow control, Unsteady flow

## Abstract

The dataset presented here regard the analysis reported in the research article entitled “Comparison of different plasma actuation strategies for aeroelastic control on a linear compressor cascade” De Giorgi et al. (2021) [1]. These data are related to the Computational Fluid Dynamics (CFD) assessment of different plasma actuation strategies for the aeroelastic control of an aero engine compressor cascade in subsonic flow conditions. The authors evaluated the accuracy of numerical computations using experimental results. Here, both experimental and raw data of the CFD simulations are presented.

## Specifications Table

**Table:** 

Subject	Aerospace Engineering, Mechanical Engineering
Specific subject area	Flow control techniques, Active Flow Control, Turbomachinery, Aeroelasticity, Aerodynamics
Type of data	Text file (Excels Data)
How data were acquired	Ansys FLUENT® R1
Data format	Raw, Analyzed
Parameters for data collection	Presented data regard a NACA65 compressor cascade in steady and unsteady flow. As performed in Motta et al. [2], inlet flow conditions are: velocity of U_∞_ = 34.36 m/s (Re≈350,000) and U_∞_ = 19.65 m/s (Re≈200,000), pressure equal to 101325 Pa, temperature equal to 288.15 K and density equal to ρ_∞_ = 1.225 kg/m^3^. Geometric model and boundary conditions employed for the numerical simulations are those real of aircraft engines compressors. Two AC voltage Dielectric Barrier Discharge (AC-DBD) plasma actuators are placed at the trailing edge of the central blades. The influence of AC-DBD plasma actuators on the aeroelastic control has been analysed through a proper phasing of the actuation switching law.
Description of data collection	Different 2D geometric domains of seven NACA65 blades were realized and imported into Ansys FLUENT® R1. Realistic operating conditions have been set and 2D Reynolds-averaged Navier-Stokes (RANS) equations have been resolved with the hypothesis of incompressible flow. Several steady runs have been executed in order to perform grid independent studies. Once the reference grid has been obtained, transient simulations of the pitching blades with and without plasma actuators have been carried out. The cosinusoidal actuation switching law for the suction and pressure side used for the assessment is as follows: $F=\left\{ \begin{array}{c} F_{\text{SS}}\;\;\text{if}\;\omega \cdot \cos\left(\omega \cdot t+n\cdot \frac{\text{IBPA}\;\cdot \;\pi }{180}+\varphi \right)\geq 0\\ F_{\text{PS}}\;\;if\;\omega \cdot \cos\left(\omega \cdot t+n\cdot \frac{\text{IBPA}\;\cdot \;\pi }{180}+\varphi \right)\leq 0 \end{array}\right.$
Data source location	University of Salento, Lecce, Italy
Data accessibility	Data available with the article
Related research article	“Comparison of different plasma actuation strategies for aeroelastic control on a linear compressor cascade” [1]

## Value of the Data

• The data are important for the since they allow to evaluate the effect of the active flow control technique based on AC-DBD Plasma Actuators in order to achieve aeroelastic control on a linear compressor cascade in unsteady flow conditions.
• The data can be useful for engineers and researchers which work in the turbomachinery research area with particular focus on active flow control.
• Data of the time history of lift and moment coefficients can be employed to verify modelling predictions of unsteady flows over a compressor cascade. Furthermore, data can be employed for comparing with the results of the application of other active flow control techniques for lift enhancement and load alleviation of a compressor cascade under unsteady flow conditions.
• Data can be used as a benchmark for future research on active flow control of unsteady flow in turbomachinery.
• This work is expected to encourage the development of AC-DBD Plasma Actuators for aeroelastic control in turbomachinery.

## 1 Data Description

**Fig. 1:**
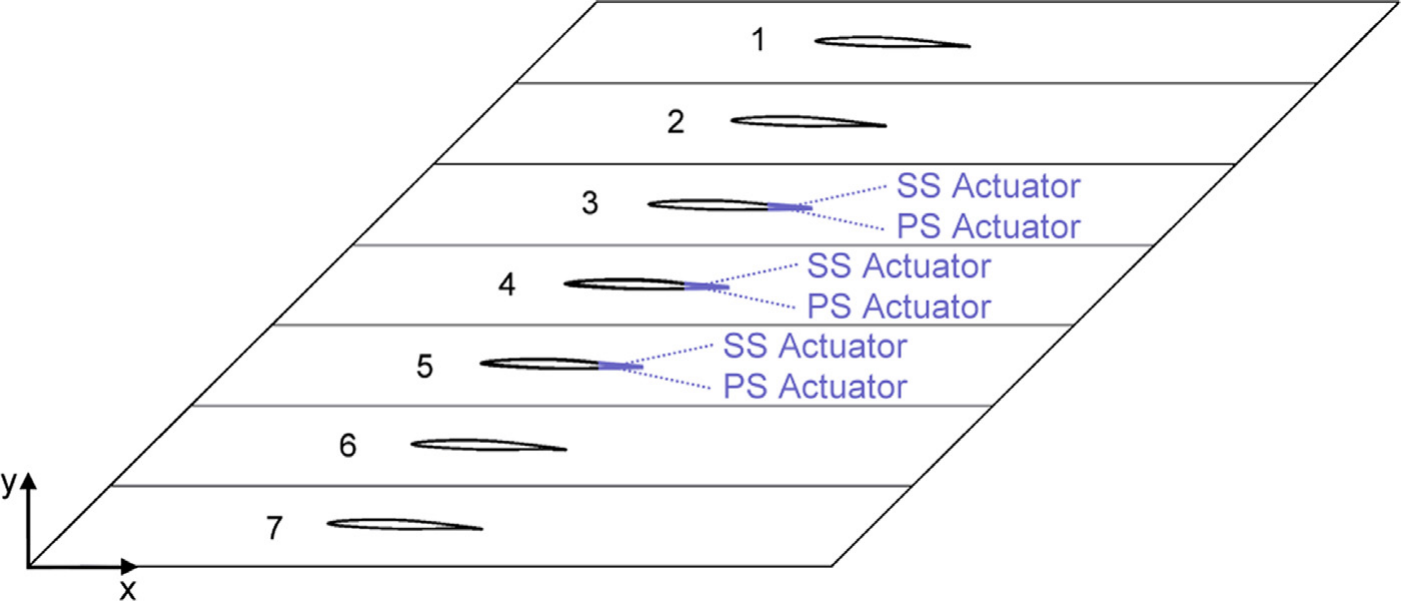
Model schematic of the subsonic compressor cascade.

**Fig. 2:**
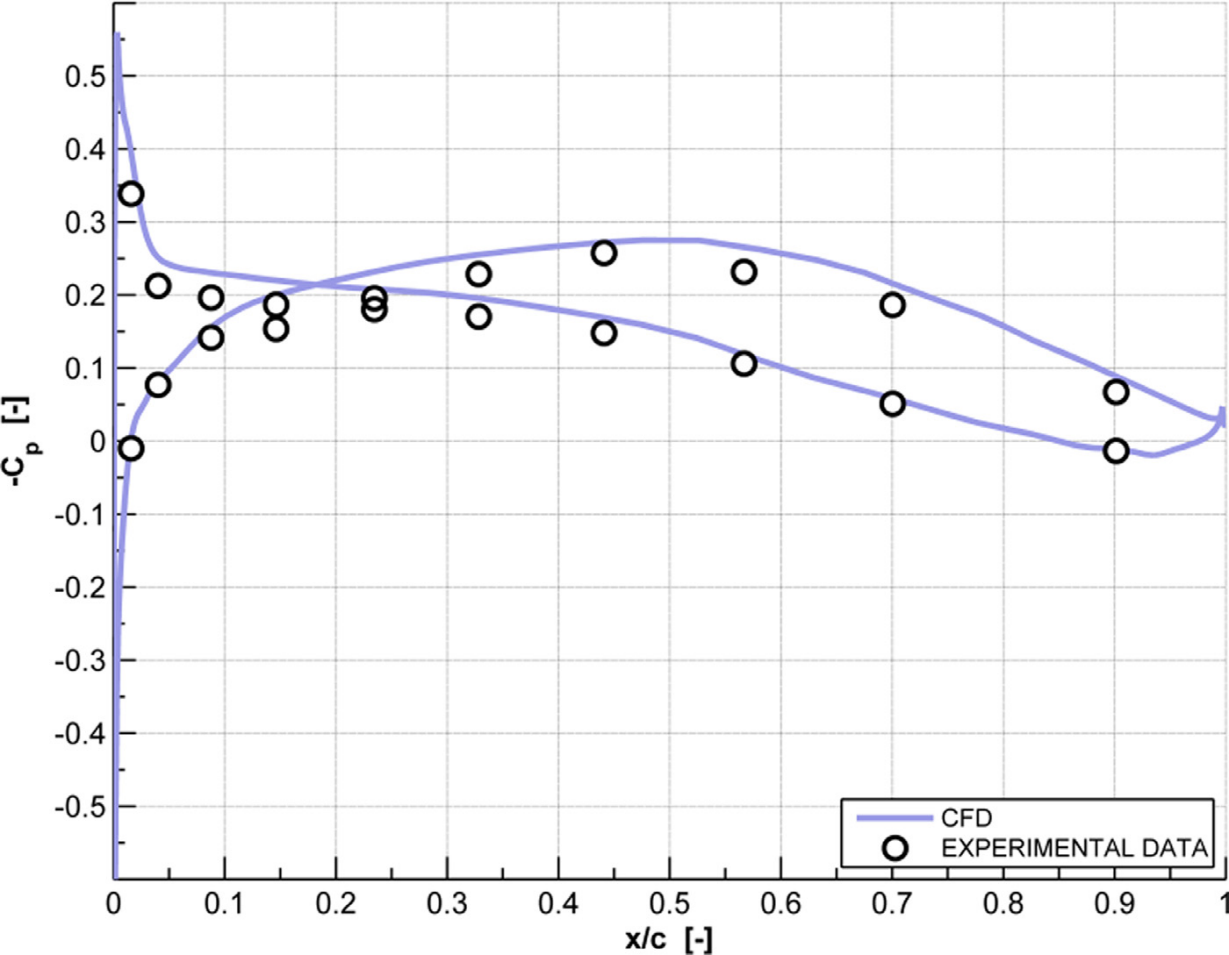
Steady pressure distribution (C_p_) of the 4th clean blade.

**Fig. 3:**
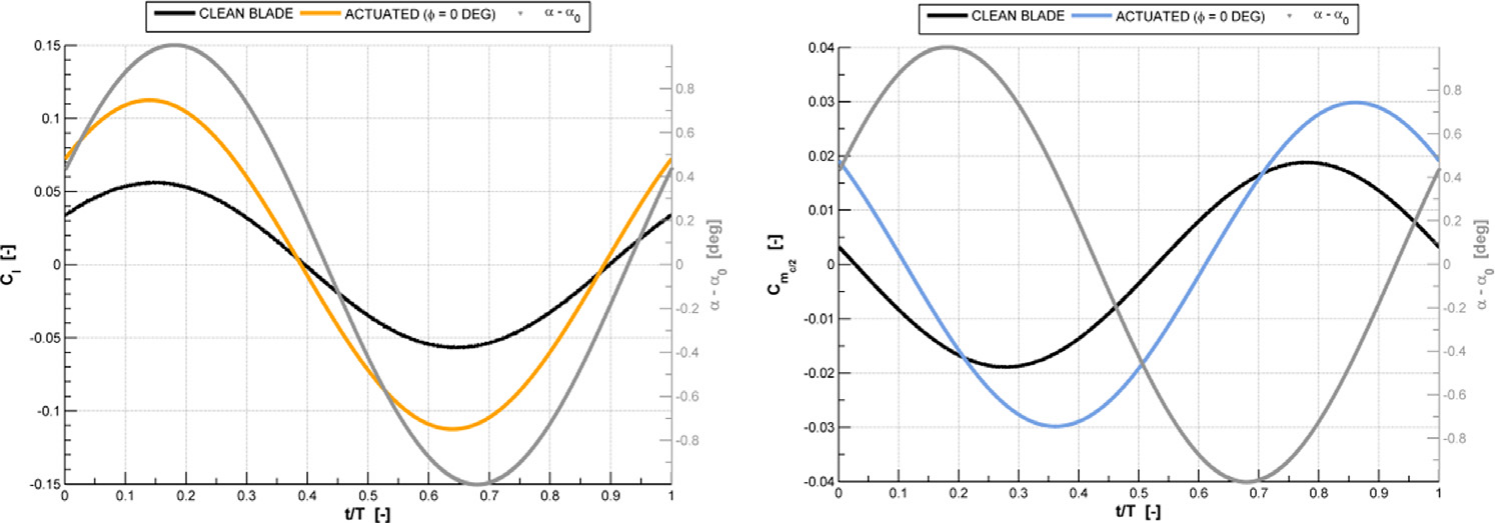
History curves of lift coefficient (C_l_) and moment coefficient (C_m,c/2_) of the 4th blade in clean and actuated configuration.

**Fig. 4:**
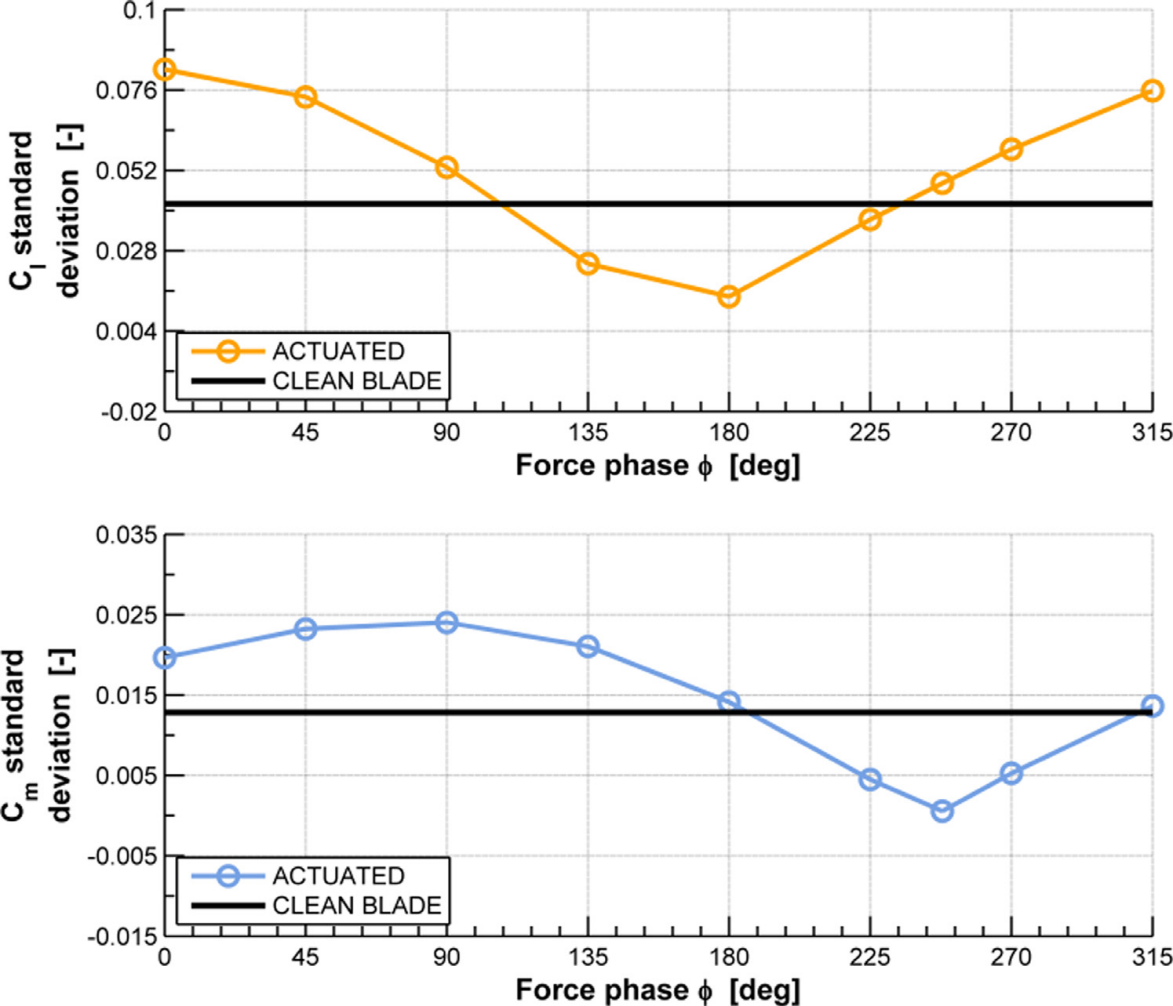
Standard deviation of lift coefficient (C_l_) and moment coefficient (C_m,c/2_) of the 4th blade in clean and actuated configuration.

The CFD dataset of a linear compressor cascade (Fig. 1) presented in the article provides the steady pressure distribution (C_p_) (Fig. 2), lift coefficient (C_l_) and moment coefficient (C_m,c/2_) time history curves (Fig. 3) and standard deviation of the 4th blade in both clean and actuated configuration (Fig. 4). Steady simulation result of the pressure coefficient is compared with the experimental data of Ref. [2], also provided in this article. As regards the C_l_ and C_m,c/2_ aerodynamic coefficients, temporal evolution of the raw signals of these coefficients in both clean and actuated configuration are reported. Furthermore, standard deviation values of these coefficients in both clean and actuated configuration are also included in this article.

In summary, the supplementary files of this article include the following data:

1. Experimental data from Motta et al. [2] and steady pressure distribution (Cp) of the 4th blade in clean configuration performed using Ansys FLUENT® R1 solver and U_∞_ = 34.36 m/s, Re≈ 350000 (Fig. 2, file “Steady Pressure Coefficient.xlsx”);
2. Comparison raw dataset between the time history of both lift and moment coefficients in clean and actuated configuration, simulated with U_∞_=19.65 m/s, Re≈200000, f=19.17 Hz, IBPA=51.43 deg and φ=0 deg (Fig. 3, file “Unsteady Lift and Moment Coefficients.xlsx”);
3. Comparison data between the standard deviation of lift and moment coefficients in clean and actuated configuration, simulated with U_∞_=19.65 m/s, Re≈200000, f=19.17 Hz, IBPA=51.43 deg and φ ∈ [0, 315] deg (Fig. 4, file “Lift and Moment Coefficients Standard Deviation.xlsx”).

## 2 Experimental Design, Materials and Methods

Subsonic steady and unsteady flow simulations around a linear compressor cascade have been performed. The 2D cascade reproduces the test facility sited at the Chair of Aero of TU Berlin [2]. The cascade comprises seven NACA65 blades with a mean angle of attack of α_0_=2 deg and chord length of c = 0.15 m. Plasma actuators (PAs) are positioned only at the trailing edge of the three central blades. The geometric domain is a 506870 elements multi-block structured quad mesh. The fluid flow is considered as incompressible, two different velocities are imposed at the inlet: U_∞_ = 34.36 m/s (Re≈350000) and U_∞_ = 19.65 m/s (Re≈200000), pressure equal to 101325 Pa, temperature equal to 288.15 K and density equal to ρ_∞_ = 1.225 kg/m^3^. Numerical computations have been carried out using the commercial solver Ansys FLUENT® R1. The k–ω turbulence model has been employed as closure for the Reynolds-averaged Navier-Stokes (RANS) equations and convergence criteria have been set to 6.35·10^−5^. Load response, evaluated in terms of lift coefficient (C_l_) and moment coefficient (C_m,c/2_) have been analysed. Pitching motion of the blades, positive clockwise, has been realized by loading a UDF macro in the solver containing the kinematic formulation of the oscillation:

(1) $$\alpha \left(t\right)=\alpha_{0}+\bar{\alpha }\text{sin}\left(\omega \cdot t+n\cdot \frac{\text{IBPA}\;\cdot \;\pi }{180}\right);$$

where α(t) is the blade angle of attack, α_0_ is the average angle of attack, ω=2πf is the frequency, n is the nth blade (starting from the top of the cascade) and IBPA is the inter-blade phase angle.

Validation of the reference grid has been carried out by comparing experimental results of Ref. [2], both in steady and unsteady flow. Dielectric barrier discharge (DBD) driven by an alternating current (AC) plasma actuator have been modelled based on Shyy's approximation [3].

The cosinusoidal actuation law employed to control the aeroelastic response of the cascade is defined as follows:

(2) $$F=\left\{ \begin{array}{c} F_{\text{SS}}\;\;\text{if}\;\omega \cdot \cos\left(\omega \cdot t+n\cdot \frac{\text{IBPA}\;\cdot \;\pi }{180}+\varphi \right)\geq 0\\ F_{\text{PS}}\;\;if\;\omega \cdot \cos\left(\omega \cdot t+n\cdot \frac{\text{IBPA}\;\cdot \;\pi }{180}+\varphi \right)\leq 0 \end{array}\right.$$

where φ indicates the force phase (in degrees) used to shift the electrical signal of the plasma actuators.

The standard deviation values of the aerodynamic coefficients signal is calculated as follows:

(3) $$\sigma =\sqrt{\frac{1}{N-1}\sum_{t=0}^{T}{\left(C_{L}\left(t\right)-C_{L,\text{mean}}\right)}^{2}};$$

## Ethics Statement

The authors followed generally expected standards of ethical behavior in scientific publishing throughout article construction.

## CRediT Author Statement

**Maria Grazia De Giorgi:** Conceptualization, Methodology, Formal analysis, Visualization, Writing – original draft. **Valentina Motta:** Supervision, Writing – review & editing. **Antonio Suma:** Supervision, Writing – review & editing. **Alessia Laforì:** Conceptualization, Visualization, Writing – original draft.

## Declaration of Competing Interest

The authors declare that they have no known competing financial interests or personal relationships which have or could be perceived to have influenced the work reported in this article.

## Appendix Supplementary materials

Image, application 1

Image, application 2

Image, application 3
